# Cryopreservation of *Plasmodium* Sporozoites

**DOI:** 10.3390/pathogens11121487

**Published:** 2022-12-07

**Authors:** Carson Bowers, Lisa Hancox, Kristen Peissig, Justine C. Shiau, Amélie Vantaux, Benoit Witkowski, Sivchheng Phal, Steven P. Maher, John T. Harty, Dennis E. Kyle, Samarchith P. Kurup

**Affiliations:** 1 Center for Tropical and Emerging Global Diseases, University of Georgia, Athens, GA 30605, USA; 2 Department of Pathology, University of Iowa, Iowa City, IA 52242, USA; 3 Department of Infectious Diseases, University of Georgia, Athens, GA 30605, USA; 4 Malaria Molecular Epidemiology Unit, Pasteur Institute of Cambodia, Phnom Penh 12201, Cambodia; 5 Department of Cellular Biology, University of Georgia, Athens, GA 30605, USA

**Keywords:** *Plasmodium*, sporozoite, cryopreservation, RAS vaccination, freezing, malaria

## Abstract

Malaria is a deadly disease caused by the parasite, *Plasmodium*, and impacts the lives of millions of people around the world. Following inoculation into mammalian hosts by infected mosquitoes, the sporozoite stage of *Plasmodium* undergoes obligate development in the liver before infecting erythrocytes and causing clinical malaria. The most promising vaccine candidates for malaria rely on the use of attenuated live sporozoites to induce protective immune responses. The scope of widespread testing or clinical use of such vaccines is limited by the absence of efficient, reliable, or transparent strategies for the long-term preservation of live sporozoites. Here we outline a method to cryopreserve the sporozoites of various human and murine *Plasmodium* species. We found that the structural integrity, viability, and in vivo or in vitro infectiousness were conserved in the recovered cryopreserved sporozoites. Cryopreservation using our approach also retained the transgenic properties of sporozoites and immunization with cryopreserved radiation attenuated sporozoites (RAS) elicited strong immune responses. Our work offers a reliable protocol for the long-term storage and recovery of human and murine *Plasmodium* sporozoites and lays the groundwork for the widespread use of live sporozoites for research and clinical applications.

## 1. Introduction

Malaria, caused by protozoan parasites of the genus *Plasmodium*, remains an unresolved global health burden that impacts over half of the world’s population [1]. Frontline artemisinin drug therapies are now threatened by the emergence of resistant parasites [2]. Despite decades of research, no globally licensed vaccines against malaria exist which provide satisfactory immunity in humans [3,4].

*Plasmodium* is transmitted through the bite of infected female Anopheles mosquitos. Mosquitoes deposit the sporozoite stage of *Plasmodium* present in their salivary glands into the skin of vertebrate hosts. Such sporozoites access the bloodstream, travel to the liver and infect hepatocytes, where they undergo obligatory replication and development into merozoites (liver-stage malaria). Mature merozoites released into circulation infect the erythrocytes and initiate the ‘blood stage’ of malaria [5]. The blood stage is responsible for the morbidity and mortality associated with malaria, and therefore, preventing the progression of *Plasmodium* to its blood stage is considered the ideal approach to vaccinating against malaria [3,6,7]. 

We have known for over fifty years that inoculation of attenuated live *Plasmodium* sporozoites, which establish abortive infections in the liver, can generate potent immune responses that protect against future challenges with virulent parasites [8,9,10]. To this day, this approach remains the gold standard for malaria vaccines and serves as the foundation for the majority of the current frontline malaria vaccination approaches [11,12,13]. The most promising of such vaccine candidates rely on attenuation through irradiation (radiation attenuated sporozoites, RAS), specific gene knockouts (genetically attenuated parasites, GAP), or chemical prophylaxis [3,14]. Regardless of how the parasites are attenuated, such approaches require the delivery of live sporozoites to vaccine recipients. However, an optimal, reliable, or transparent technology to preserve live sporozoites for clinical use or research purposes does not currently exist.

Historically, cryopreservation has been used to preserve and extend the shelf lives of eukaryotic cells [15,16]. However, freezing itself can have detrimental effects on the structure or function of cells [17,18], and is even considered an effective method for killing or inactivating sporozoites in certain cases [19]. Therefore, optimal cryopreservation requires the use of suitable cryoprotectants and protocols that preserve the vitality of the samples. Early attempts to cryopreserve sporozoites included suspending disrupted salivary glands from infected mosquitoes in host serum and freezing them [20] or freezing whole infected mosquitos [21]. There has been some success with the preservation of sporozoites using host serum and certain cryoprotective additives [22,23]. Commercially available cryopreservatives have also shown some efficacy in the preservation of sporozoites [24]. Some commercial organizations have claimed success in preserving and recovering sterile sporozoites, although the precise methodologies employed remain unknown and the results inconsistent [25,26]. Our goal was to develop a broadly available and transparent method for the reliable, consistent, and extended preservation of *Plasmodium* sporozoites. 

In this study, we describe a novel cryopreservation reagent and outline a protocol for long-term cryopreservation and recovery of the sporozoites of both human and rodent malaria parasites. Using in vitro and in vivo studies, we demonstrate that the recovered *Plasmodium* sporozoites can infect hepatocytes and progress to blood-stage infection in vivo. We also show that cryopreserved RAS can induce strong immune responses in mice, offering support for the application of our cryopreservation strategy in research on human malaria vaccines.

## 2. Materials and Methods 

### 2.1. Mice and Parasites 

C57BL/6 (B6) mice were procured from Jackson Laboratories and were bred and housed with appropriate biosafety containment at the animal care unit at the University of Georgia (UGA). The mice were handled in accordance with the guidelines established by the UGA Institutional Animal Care and Use Committees. Mice of either sex and 4–6 weeks of age were used to initiate the studies. 

*P. yoelii* (17XNL) was obtained from BEI resources (MRA-593). *P. falciparum* (BD007) was originally isolated from Bangladesh and maintained at UGA [27]. Mosquitoes bearing wild-type *P. berghei* or *Pb-Luc-GFP* were purchased from UGA SporoCore. The sporozoite stages of these parasites were obtained by infecting *Anopheles stephensi* mosquitoes at the insectaries at UGA or the University of Iowa and isolated using established protocols [27,28]. Infected mosquitoes were kept at 24 °C ± 5 °C, 80% ± 5% relative humidity, and under a 12-h day/night photoperiod schedule (Percival Scientific, Perry, IA, USA) with 5% dextrose (*w*/*v*) and 0.05% para-Aminobenzoic acid (*w*/*v*) (Thermo Fisher, Hampton, NH, USA). *P. falciparum* sporozoites were generated at UGA SporoCore as described in detail before [27]. *P. vivax* sporozoites were generated from human patient isolates from Cambodia using *Anopheles dirus* mosquitos as previously described [29]. It should be noted that the *Plasmodium* species infecting mice and humans are fundamentally different in their biology, and the protocols for isolation of sporozoites and in vivo infections vary depending on species as outlined below. 

### 2.2. Determining Sporozoite Integrity and Viability 

Sporozoites were isolated from infected mosquitoes as described before [30]. In brief, mosquito salivary glands were sheared using a 30-gauge needled syringe to release the sporozoites. Sporozoites were counted using a hemocytometer (incyto, Chungnam-do, South Korea) before irradiating, inoculating into cultures or mice, or undergoing cryopreservation. Counts of thawed, cryopreserved sporozoites were compared to the initial number of sporozoites frozen to assess the retention of the structural integrity of sporozoites. To determine viability, the sporozoites were stained with propidium iodide (PI) and counted as described before [31]. In short, freshly isolated or cryopreserved sporozoites were reconstituted with 10X PI and the frequency of viable sporozoites (PI stain negative) was enumerated using fluorescence microscopy. 

### 2.3. In Vitro and In Vivo P. berghei and P. yoelii Sporozoite Infections

1 × 10^6^ Hepa 1-6 hepatocyte cells resuspended in DMEM (Sigma, St. Louis, MO, USA) with 10% *v/v* FCS (Sigma) were plated in 40-mm glass-bottom culture dishes (Greiner bio-one, Monroe, NC, USA, at 5 × 10^5^ cells/well) for at least 4 h before inoculation with 1 × 10^4^ freshly isolated or cryopreserved *P. berghei* sporozoites. The infected cultures were washed twice with PBS and imaged at 36 h post-infection (p.i.) (Applied Precision DeltaVision Microscope System, GE Healthcare, Issaquah, WA, USA) [27]. Cryopreserved *Pb-Luc-GFP* parasites were visualized using the GFP channel to assess conservation of transgenic properties following cryopreservation.

For in vivo infections, 1 × 10^3^ freshly isolated or cryopreserved *P. berghei* or *P. yoelli* sporozoites were enumerated and resuspended in 200 µL complete normal saline before inoculating into B6 mice intravenously (i.v.). The parasite burdens or immune responses were assessed at 24 h, 44 h, or 7–10 days p.i.

### 2.4. Flow Cytometry

Parasitemia was assessed by flow cytometry as described before [32]. In short, at the indicated time points, whole blood samples were collected from mice through tail-bleeding, and stained with Phycoerythrin (PE) conjugated anti-Ter119 (Tonbo Biosciences, San Diego, CA, USA). Data was acquired on a Quanteon flow cytometer (NovoCyte, Santa Clara, CA, USA) and analyzed using FlowJo X (Treestar, Ashland, OR, USA). The frequency of *Pb-GFP-Luc* infected RBCs was ascertained by their GFP signal. To assess the immune responses generated following immunization, blood cells were stained using GAP50 tetramer (APC, NIH tetramer core, Atlanta, GA, USA), anti-CD8a (PEcy7, BioLegend, San Diego, CA, USA), or anti-CD11a (APC, BioLegend) as described before. CD11a^hi^CD8^lo^ cells indicate the activated fraction of CD8 T cells and GAP50^+^ fraction represents *Plasmodium*-specific activation of the CD8 T cells [30].

### 2.5. Luminescence Signal Imaging 

Cryopreserved *Pb-GFP-Luc* parasites were detected in tissue through bioluminescence imaging, as described before [33]. In short, prior to bioluminescent imaging, the mice were anesthetized with 2% isoflurane and then injected with 200 µL (150 µg/mL in PBS) of luciferin substrate (Syd Labs, Hopkinton, MA, USA), through the intraperitoneal (i.p.) route. The luciferin substrate was allowed to adequately disseminate in the mice for 10 min before imaging. Mice were imaged using a bioluminescent imaging system (IVIS 100; Xenogen, Alameda, CA, USA). Mice were placed in the imaging chamber, where a controlled flow of 1.5 % isoflurane was administered through the nose cone of a gas anesthesia system. Mice were imaged in dorsal recumbency, the images were collected using a 5 min integration time and analyzed using the Living-Image software (Xenogen) where luminescence is quantified as the sum of all detected photon counts per second.

### 2.6. Primary Human Hepatocyte Culture for P. falciparum and P. vivax Infection

Primary human hepatocyte infection in this study followed published methods [34]. Briefly, cryopreserved primary human hepatocytes (BioIVT, Westbury, NY, USA) were thawed two days prior to infection and seeded with 1.8 × 10^3^ cells per well in a 384-well collagen-coated plate (Greiner Bio-One). Hepatocyte culture was maintained with daily media change with customized InVitroGro HI medium without dexamethasone (BioIVT) supplemented with 5% human serum (Interstate Blood Bank, Memphis, TN, USA), 1:100 dilution of penicillin/streptomycin/neomycin antibiotic mixture (Gibco, Hampton, NH, USA), and 10 μg/mL gentamicin (Gibco). 1 × 10^3^ sporozoites were inoculated into the hepatocyte culture, and the plate was centrifuged at 250 g, at room temperature, and for 5 min. Hepatocyte cultures were kept at 38 °C with 5% CO_2_ in an incubator (Panasonic, Kadoma, Osaka, Japan).

### 2.7. P. falciparum and P. vivax Sporozoite Infection and Quantification

At 4 days post-infection, infection levels were assessed using immunofluorescence microscopy [35]. Hepatocyte cultures were fixed with 4% paraformaldehyde (Alfa Aesar, Hampton, NH, USA) for 10 min at room temperature and washed twice with 1×PBS followed by permeabilization, blocking, and staining with 0.03% Triton-X (Acros, Hampton, NH, USA), 1% BSA (*w*/*v*) (FisherSci, Hampton, NH, USA) and 1 μg/mL mouse anti-GAPDH 13.3 (European Malaria Reagent Repository) overnight at 4 °C. The secondary stain was applied using anti-mouse AF-488 (Thermo Fisher) overnight at 4 °C. The wells were then stained with 10 µg/mL Hoechst under room temperature for 1 h. Fixed and stained samples were stored in PBS for quantification. 

The parasites in each well were imaged using ImageXpress (Molecular Devices, San Jose, CA, USA). Images were acquired using FITC and DAPI channels at 10X magnification with a total of nine fields of view per well. The infection statuses of individual wells were assessed by stitching together the nine images acquired from each field of view. 

For *P. vivax*, at 8 days post-infection, infection levels were assessed using an immunofluorescence assay as described above. Hepatocyte cultures were fixed, permeabilized, blocked, and stained as above, but using anti- *P. vivax* UIS4 specific antibody [36]. The parasites in each well were imaged using a Lionheart FX (Agilent, Santa Clara, CA, USA). Images were acquired at 4X magnification with a total of 4 fields of view per well. 

### 2.8. Radiation Attenuated Sporozoite (RAS) Vaccination 

Sporozoites were gamma-irradiated (200Gy) as described before [37] and cryopreserved. Following the recovery of cryopreserved RAS, 1 × 10^4^ freshly isolated sporozoites or RAS were injected intravenously into B6 mice. On day 7 post-inoculation, 20 µL of blood from the vaccinated or control mice was collected for analysis. 

### 2.9. Statistical Analysis 

Statistical comparisons between two groups were performed using two-tailed t-tests and performed using Prism 9 software (GraphPad, San Diego, CA, USA). *p* > 0.05: n.s., *p* ≤ 0.05: *.

## 3. Results

### 3.1. Cryopreservation of Plasmodium Sporozoites

In order to establish a methodology for long-term storage and recovery of *Plasmodium* sporozoites, we developed a novel cryopreservation protocol utilizing freezing media with two distinct fractions (Table 1). The key difference between fractions A and B is that fraction B contains the low molecular weight (LMW) cryoprotectant, glycerol. Glycerol minimizes intracellular damage due to water crystal formation during the freeze–thaw process [38]. Both fractions contain citric acid (anhydrous, Sigma-Aldrich, St. Louis, MO, USA), which acts as a preservative and antioxidant, as well as glucose, which acts as a source of energy and as a secondary cryoprotectant. When prepared for mouse or human malaria parasites, the cryopreservation media would be formulated with the mouse or human sera, respectively. 

**Figure 1:**
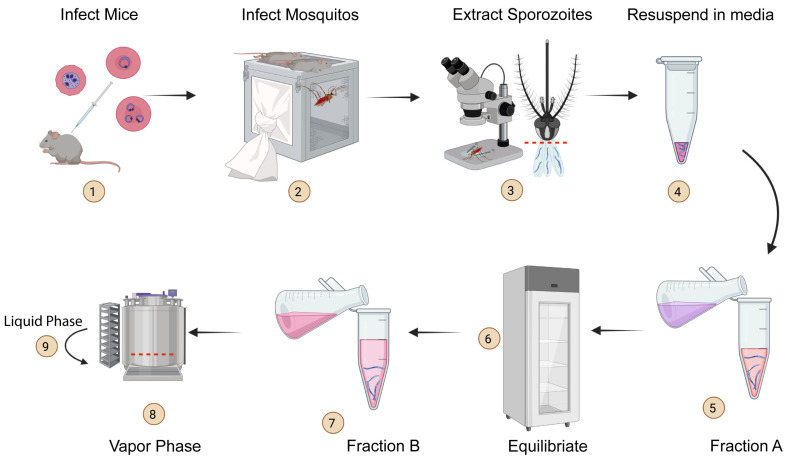
**Cryopreservation protocol for *Plasmodium* sporozoites.** Mice are infected with the murine *Plasmodium* spp. of interest (**1**). Once parasitemia reaches 2–4%, female mosquitos (*Anopheles stephensi*) are allowed to feed on the infected mice to transfer the infection (**2**). In the case of human malaria parasites, mosquitos are fed on parasitized red blood cells instead. The infected mosquitos are dissected and salivary glands removed (**3**), the glands disrupted using a 30-gauge syringe and resuspended in 100 µL media containing 1% mouse serum (**4**). An equal volume (100 μL) of Fraction A (Table 1) of the freezing medium is added to the isolated sporozoites in media (**5**) and equilibrated at 4 °C for 2 h (**6**). An equal volume of cold (4 °C) Fraction B (Table 1, 200 μL) is added to the tube containing the sporozoites with Fraction A (**7**). The sporozoites in the media (400 μL) are subsequently frozen down in the vapor phase of LN2 (**8**). After at least 30 min, the tubes containing sporozoites are transferred to the liquid phase of LN2 for long-term storage (**9**).

We initially optimized the cryopreservation protocol using the mouse malaria parasites, *P. berghei* and *P. yoelii*, primarily owing to practical considerations, such as their relative ease of availability, biosafety, and the technical simplicity of testing the recovery of cryopreserved samples in vitro and in vivo. As outlined in Figure 1, the cryopreservation protocol starts with the isolation of fresh sporozoites from *Plasmodium*-infected female *A. stephensi* mosquitoes into the sporozoite resuspension media (Table 1). After isolating the salivary glands from mosquitoes, the salivary glands were disrupted, the sporozoites enumerated, and 1 × 10^4^–1 × 10^5^ sporozoites resuspended in a cryovial containing 100 µL of the sporozoite resuspension media (Table 1). An equal part (100 µL) of pH-adjusted fraction A of sporozoite freezing media (Table 1) was then added to the cryovial and equilibrated at 4 ℃ for 2 h. An equal part of fraction B (200 µL), also at 4 ℃, was then added to the equilibrated sporozoites resulting in a final volume of 400 µL. The cryovials containing the chilled sporozoites were then placed in the vapor phase of liquid nitrogen (LN_2_) in a freezer for rapid freeing. After 30 min in the vapor phase, the cryovials were transferred into the liquid phase of LN_2_ for prolonged storage (Figure 1). 

To recover the cryopreserved sporozoites, the cryovials drawn from LN2 were immersed in water at room temperature so that the contents were rapidly and completely thawed. The samples were immediately diluted in media, and centrifuged with no brake at 6000g for 10 min at room temperature (RT). The supernatant was carefully discarded, and the pellet resuspended in normal saline or media as suitable for in vitro or in vivo applications. 

### 3.2. The Metrics of Successful Cryopreservation and Recovery

We evaluated the efficiency of our cryopreservation protocol by quantifying three distinct attributes: integrity, viability, and infectiousness. We compared these values in sporozoites before and after cryopreservation and recovery. Integrity indicates the retention of the shape and form of the sporozoites following cryopreservation and recovery. This is calculated by comparing the sporozoite counts before and after cryopreservation in each sample vial, using light microscopy. Cryopreservation is known to result in cellular damage due to changes in osmotic pressure and water crystal formation [18,39]. Therefore, integrity is a valuable metric to determine the extent of sporozoite loss due to freezing injury. Viability represents the proportion of sporozoites that retain intact plasma membranes following cryopreservation, as determined by a dye-exclusion assay using propidium iodide (PI). Viability is expressed as the percentage of PI unstained (PI negative) of the total sporozoites present in the sample [31]. 

Access to cryopreserved sporozoites can have a significant impact on basic and translational research. It is imperative that the sporozoites endure the cryopreservation process and reproducibly infect hepatocytes in order to serve as a tool for such studies. In the case of mouse malaria parasites, infectiousness was assessed in vivo and expressed as the frequency of mice that exhibited blood-stage infection following inoculation with the 1 × 10^3^ sporozoites. In the case of human malaria parasites, in vivo assessment of progression to blood-stage malaria is ethically limited. Although the use of liver-humanized mice is a possibility, this too is logistically challenging [18,39]. Therefore, infectiousness in human malaria parasites was assessed in vitro 4 d.p.i and represented the ability of the recovered cryopreserved sporozoite samples to establish an infection in primary human hepatocyte cultures. Infectiousness represents the percentage of wells in a 384-well plate with 1.8 × 10^3^ hepatocytes that were infected following the addition of 1 × 10^3^ sporozoites/well.

### 3.3. Effective Cryopreservation and Recovery of Plasmodium Sporozoites

**Figure 2:**
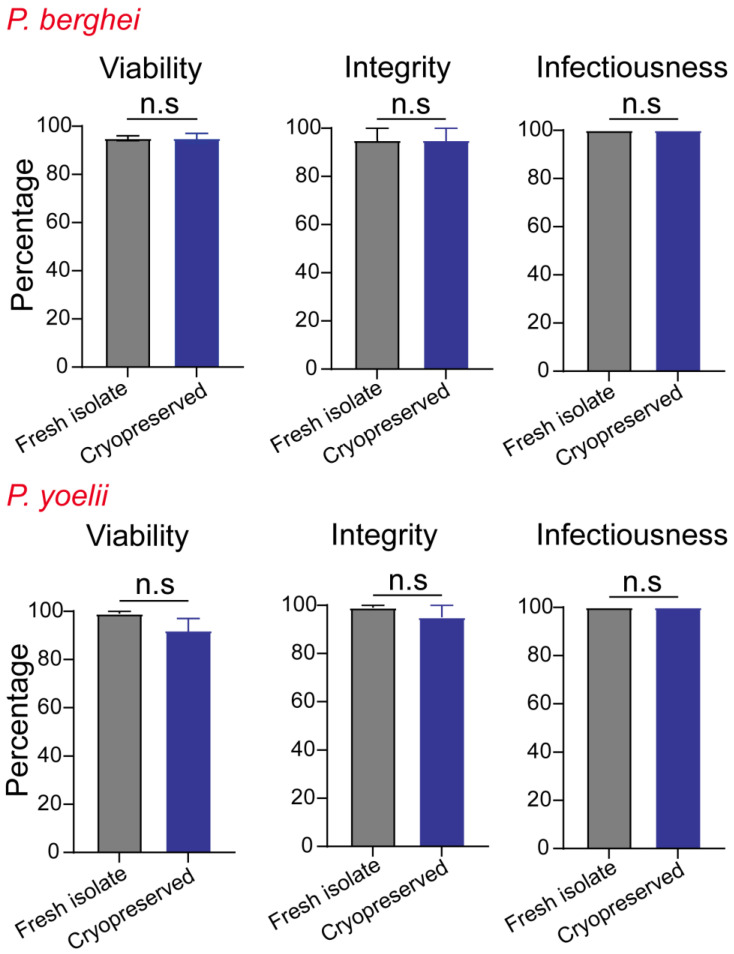
**Cryopreservation retains the vitality of *P. berghei* and *P. yoelii* sporozoites.** Bar graphs depicting the integrity, viability, and infectiousness of freshly isolated or cryopreserved (9 months) wild type *P. berghei* and *P. yoelii* sporozoites. All data collected from in vitro (integrity, viability) or in vivo (infectiousness) assays with at least three technical or biological replicates. Infectiousness indicates the frequency of mice exhibiting blood-stage infection following inoculation with 1 × 10^3^ freshly isolated or cryopreserved sporozoites at 7–10 d.p.i. Data presented as mean+ s.e.m and analyzed using 2-tailed t-tests, n.s = *p* > 0.05.

**Figure 3:**
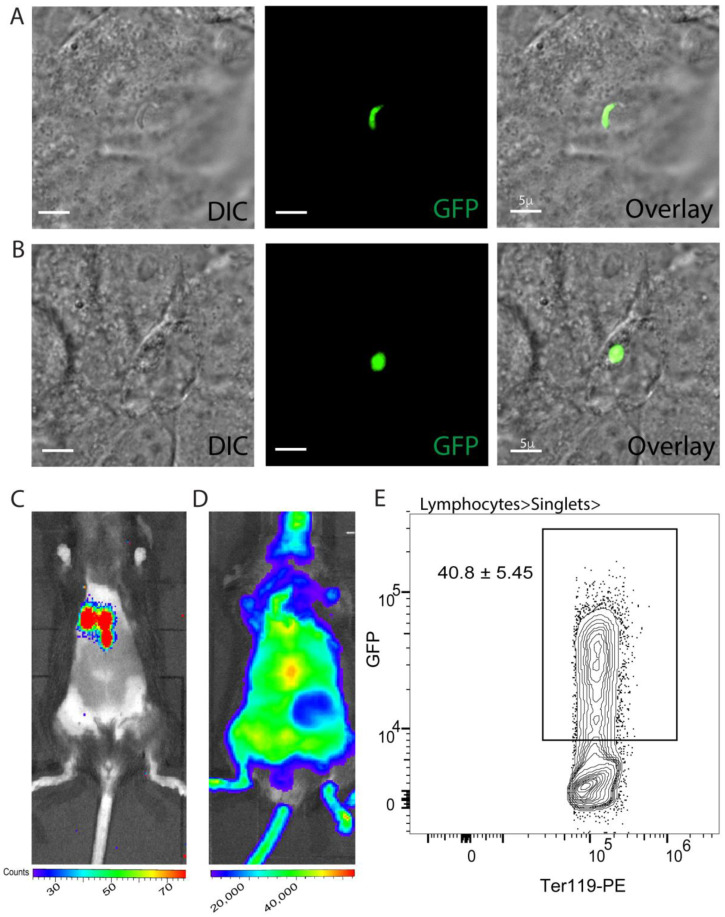
**Cryopreservation retains infectiousness and function in sporozoites.** (**A**,**B**) Microscopy images showing different developmental stages- sporozoites (**A**) and the extra-erythrocytic form in development (**B**) in in vitro cultured hepatocytes in recovered *Pb-GFP-Luc* after cryopreservation for 14 days. DIC: Differential Interference Contrast image. (**C**) Representative image depicting luminescence signal detected in B6 mice inoculated with cryopreserved *Pb-GFP-Luc* sporozoites (5 × 10^4^/mouse), 44 h post-inoculation. (**D**) Representative image depicting luminescence signal detected in B6 mice inoculated with cryopreserved *Pb-GFP-Luc* sporozoites (5 × 10^4^/mouse), 7 days post-inoculation. (**E**) Representative flow plot indicating the frequency of infected RBCs in a B6 mouse inoculated with cryopreserved *Pb-GFP-Luc* sporozoites (5 × 10^4^/mouse), 10 days post-inoculation. Data presented as mean + s.e.m from two separate replicate experiments, with 3 mice per group.

Both *P. berghei* and *P. yoelii* sporozoites retained high levels of integrity, viability, and infectiousness following cryopreservation for up to 9 months (Figure 2). We observed no statistically significant differences in the integrity of sporozoites present in cryopreserved samples when compared to the samples of *P. berghei* or *P. yoelii* before cryopreservation. Similarly, we found no significant loss of viability following the cryopreservation of *P. berghei* or *P. yoelii* sporozoites, and the samples exhibited 100% infectiousness in mice (Figure 2 and Figure 3). 

To evaluate the extent to which cryopreservation would impact the transgenic properties of *Plasmodium* sporozoites following cryopreservation, we determined the expression of green fluorescent protein (GFP) and luciferase activity in the cryopreserved transgenic *P. berghei* encoding the GFP and the firefly luciferase genes (*Pb-GFP-Luc*). GFP expression was visualized using microscopy in *Pb-GFP-Luc* sporozoites or *Pb-GFP-Luc* infected primary hepatocyte cultures. Luciferase activity was determined by the ability of *Pb-GFP-Luc* to break down D-Luciferin in vivo in infected mice. We observed clear, detectable GFP and luciferase signals in vitro and in vivo respectively following infection with the cryopreserved *Pb-GFP-Luc* sporozoites (Figure 3A–C). The continued expression of GFP or luciferase by *Pb-GFP-Luc* in the above experiment implied that vital cell functions related to the expression and function of proteins are preserved in sporozoites following cryopreservation using our protocol. Genetic attenuation through transgenesis is a frontline approach to making sporozoite-based vaccines against malaria today [3] and therefore, the retention of genetic changes through cryopreservation is a critical consideration. Of note, GFP and luciferase activity were also retained through to the blood stage initiated by the cryopreserved *Pb-GFP-Luc* sporozoites (Figure 3D, E). 

We next tested the efficacy of our cryopreservation protocol for *P. falciparum* and *P. vivax* parasites, which are the major *Plasmodium* species infecting humans. The sporozoites of either species remained fully infectious, although the integrity and viability values were impacted by cryopreservation (Table 2). Of note, freshly isolated sporozoites of *P. falciparum* and *P. vivax* typically show >90% viability and 100% integrity and infectiousness (data not shown). We determined the infectiousness of *P. falciparum* and *P. vivax* sporozoites in vitro by inoculating primary human hepatocyte cultures.

### 3.4. Immunization with Cryopreserved Radiation Attenuated Sporozoites 

**Figure 4:**
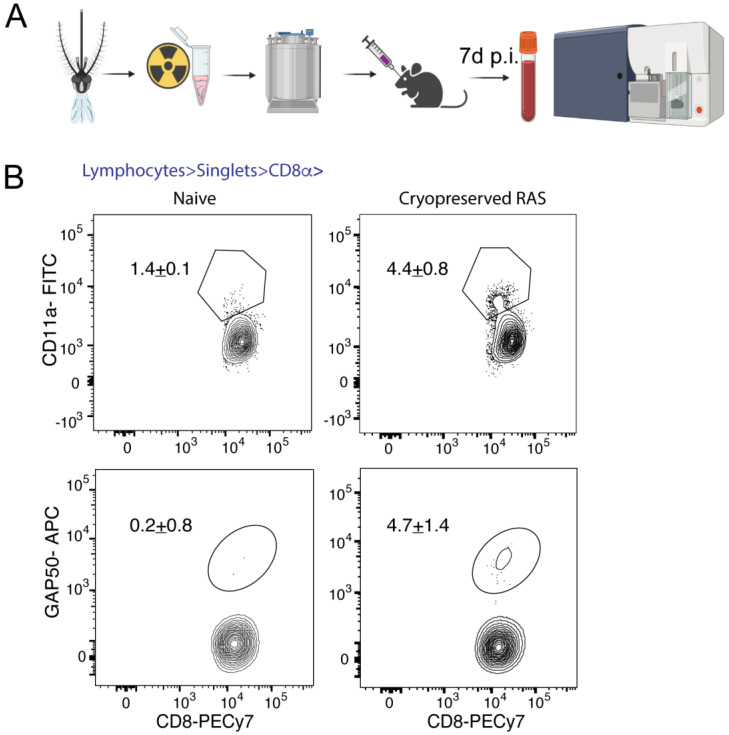
Cryopreserved radiation-attenuated sporozoites (RAS) induce strong immune responses. (**A**) Schematic depicting the generation of cryopreserved RAS vaccine. *Plasmodium* sporozoites isolated from freshly dissected mosquitos are attenuated through irradiation and preserved using the protocol outlined in Figure 1. Recovered sporozoites are inoculated into mice intravenously. Seven days after RAS vaccination, blood is collected and screened by flow cytometry. (**B**) Representative flow plots showing the frequency of activated (CD11a^hi^CD8^lo^) CD8 T cells (upper panel) or GAP50 epitope-specific CD8 T cells (lower panel) in B6 mice following vaccination with 1 × 10^4^ cryopreserved *P. yoelii* RAS, at 7-day post inoculation, presented as percentages. Naïve mice served as controls. Data represent two biological replicate experiments with at least 3 mice per group and presented as mean + s.e.m. The mean frequencies of activated and GAP50-specific CD8 T cell frequencies in B6 mice immunized with freshly isolated RAS were respectively 14.0 ± 4.5% and 2.26 ± 0.8%.

One area where reliable cryopreservation of *Plasmodium* sporozoites is crucial is the long-term storage and transportation of whole sporozoite-based vaccines for malaria. Therefore, we sought to determine how effective our protocol is for the long-term preservation and recovery of radiation attenuated sporozoites (RAS) by determining the ability of cryopreserved RAS to induce immune responses in mice. Of note, irradiation itself did not alter the integrity of viability of the cryopreserved sporozoites (data not shown). We inoculated fresh or cryopreserved *P. yoelii* RAS into B6 mice and determined the ability to prime *P. yoelii*-specific CD8 T cell responses in mice. Of note, CD8 T cells are the primary mediators of protective immunity following immunization with live-attenuated sporozoite-based vaccines such as RAS [40,41,42]. When *P. yoelii* RAS cryopreserved for nine months were recovered and inoculated into B6 mice (Figure 4A), they induced strong *Plasmodium*-specific CD8 T cell responses (Figure 4B). This suggested that our cryopreservation approach can be potentially used to preserve live-attenuated sporozoite-based vaccines.

## 4. Discussion

In this study, we established a protocol for the cryopreservation of *Plasmodium* sporozoites. We determined how our protocol impacted the fitness of the recovered sporozoites through defined metrics such as integrity, viability, and infectiousness. We found that cryopreservation in *P. berghei, P. yoelii,* and *P. falciparum* sporozoites had minimal impact on their integrity, viability, and infectiousness, while both integrity and viability, but not the infectiousness of *P. vivax* sporozoites were compromised to a certain extent. We also showed that sporozoites maintained their transgenic properties through the cryopreservation process and that radiation attenuated cryopreserved sporozoites successfully generated *Plasmodium*-specific immune responses following the immunization of mice with them. 

Previous attempts at preserving *Plasmodium* have shown varying degrees of success over the years [21,24,43,44,45], although there is yet to be found a widely accepted method of preservation for *Plasmodium* parasites outside of the blood stage. Most commonly, cryopreservation of sporozoites involves resuspending sporozoites in media containing serum or salt solution (HBSS) [23,44] with the addition of cryopreservatives such as hydroxyethyl starch [22], DMSO [46], or glycerol [47]) and storage at −80 or in LN2 [48,49]. Though fetal calf serum (FCS) is commonly utilized to reconstitute the cryopreservation media [24,46], our preliminary observations (data not shown) indicate that the use of host serum (mouse or human, as applicable), as in some of the published studies [22,48] is more optimal for retaining the fitness of cryopreserved sporozoites. 

Few studies have comprehensively evaluated the various metrics of parasite fitness such as viability or included determinations of in vitro or in vivo infectivity. In some cases, proxy measures for infectiousness or vigor, such as gliding motility have been used [24,45]. In the rare cases where the ability of the cryopreserved parasites to infect hepatocytes have been examined, it was looked at in isolation and without correlates of parasite fitness such as viability [23,48], and has shown a wide range of efficacies. In some instances, infectiousness has been determined following cryopreservation with proprietary formulations or protocols which are not publicly available [43,49,50]. We have outlined a comprehensively tested protocol for preserving infectious *Plasmodium* sporozoites, which would be available in the public domain for continued testing and widespread application. Of note, we have observed that adherence to our optimized cryopreservation protocol is just as important as the composition of the cryopreservation media for the success of the process.

Cryopreserved *P. falciparum* consistently outperformed *P. vivax* in integrity and viability measures. While the reasons for this remain unknown, it is noteworthy that *P. vivax* sporozoites in our experiments were derived from mosquitoes fed on *P. vivax* isolated from patients. The *P. falciparum* sporozoites were, in comparison, collected from mosquitoes infected with parasites maintained routinely in culture. The uniformity of the fitness levels of either parasite line may be vastly different. Future optimization of the *P. vivax* cryopreservation media and protocol may further enhance the efficiency of its cryopreservation.

Research using *P. vivax* sporozoites is often limited to field sites where *P. vivax* is endemic. This is primarily because *P. vivax* is notably difficult to culture in vitro [51,52] or preserve [46] and researchers rely on the isolation of these parasites from patients in endemic regions. Our cryopreservation protocol, albeit limited in efficacy, allows for the preservation and transportation of *P. vivax* sporozoites to study sites outside of endemic areas, broadening the avenues for research on *P. vivax* biology. 

Through this work, we have provided a framework for cryopreserving and assessing the efficacy of cryopreservation of *Plasmodium* parasites for use in malaria research and vaccine development. Although the mouse malaria parasites are more pertinent to basic research studies, the human malaria parasites have major public health significance. Widespread use of our protocol for sporozoite cryopreservation will allow a larger pool of researchers to access viable sporozoites that can initiate liver-stage infections. We believe that our work also lays the foundation to expand access to sporozoites for clinical applications, such as live-attenuated malaria vaccines for use in humans.

## Figures and Tables

**Table 1:** Components of two-fraction sporozoite freezing medium. Columns indicate the amount (weight or volume) of each component added to either fraction A or fraction B of the freezing medium. The intended function of each component is also shown. LMW: low molecular weight.

Component	Fraction A (pH 7 with NaOH or HCl)	Fraction B (pH 7 with NaOH or HCl)	Purpose
Citric Acid	1.34 g	1.34 g	Preservative, antioxidant, metabolite
Glucose	1 g	1 g	Sugar, secondary LMW cryoprotectant
Glycerol	none	14 mL	Primary LMW cryoprotectant
Sporozoite media *	20 mL	20 mL	Antibiotics, eukaryotic cell protective proteins
Sterile Water	To make 100 mL	To make 100 mL	Solvent

* Sporozoite resuspension media: DMEM (Sigma; D5671) with 1% mouse or human (heat-inactivated) serum as applicable, with penicillin and streptomycin (Sigma P0781; 1% *v/v*).

**Table 2:** Metrics of cryopreservation in human *Plasmodium* species. Values are presented as mean ± SEM (n = 3) following 14 days of cryopreservation. Infectiousness was determined by the addition of 1 × 10^3^ cryopreserved sporozoites into a well with 1.8 × 10^3^ primary human hepatocytes. Infectiousness indicates the frequency of hepatocyte cultures that contained *Plasmodium* infected hepatocytes at 4 d.p.i. Please note that human malaria parasites do not infect hepatocytes of murine origin.

Strain	Integrity	Viability	Infectiousness
*P. falciparum*	95 + 1.22	84.3 ± 8.0	100 ± 0.0
*P. vivax*	66.35 ± 0.12	44.17 ± 7.6	100 ± 0.0

## Data Availability

All primary data are available from the corresponding author upon request.
